# Heterozygous TLR3 Mutation in Patients with Hantavirus Encephalitis

**DOI:** 10.1007/s10875-020-00834-2

**Published:** 2020-09-16

**Authors:** Terhi Partanen, Jie Chen, Johanna Lehtonen, Outi Kuismin, Harri Rusanen, Olli Vapalahti, Antti Vaheri, Veli-Jukka Anttila, Michaela Bode, Nina Hautala, Tytti Vuorinen, Virpi Glumoff, Minna Kraatari, Pirjo Åström, Janna Saarela, Heikki Kauma, Lazaro Lorenzo, Jean-Laurent Casanova, Shen-Ying Zhang, Mikko Seppänen, Timo Hautala

**Affiliations:** 1 Department of Internal Medicine, Oulu University Hospital, Oulu, Finland; 2 St. Giles Laboratory of Human Genetics of Infectious Diseases, Rockefeller Branch, The Rockefeller University, New York, NY 10065 USA; 3 Institute for Molecular Medicine Finland, HiLIFE, University of Helsinki, Helsinki, Finland; 4 The Folkhälsan Research Center and Medicum, University of Helsinki, Helsinki, Finland; 5 Department of Clinical Genetics, Oulu University Hospital, Oulu, Finland; 6 Department of Neurology, Oulu University Hospital, Oulu, Finland; 7 Department of Virology, University of Helsinki and HUS Helsinki University Hospital, Helsinki, Finland; 8 Department of Infectious Diseases, Inflammation Center, University of Helsinki and HUS Helsinki University Hospital, Helsinki, Finland; 9 Department of Diagnostic Radiology, Oulu University Hospital and University of Oulu, Oulu, Finland; 10 Department of Ophthalmology, PEDEGO Research Unit, Medical Research Center, Oulu University Hospital and University of Oulu, Oulu, Finland; 11 Department of Medical Microbiology, Institute of Biomedicine, Turku University Hospital and University of Turku, Turku, Finland; 12 Research Unit of Biomedicine, University of Oulu, Oulu, Finland; 13 Centre for Molecular Medicine Norway, University of Oslo, Oslo, Norway; 14 Paris Descartes University, Imagine Institute, 75015 Paris, France; 15 Laboratory of Human Genetics of Infectious Diseases, Necker Branch, INSERM UMR 1163, Necker Hospital for Sick Children, 75015 Paris, France; 16 Pediatric Hematology-Immunology Unit, Necker Hospital for Sick Children, 75015 Paris, France; 17 Howard Hughes Medical Institute, New York, NY 10065 USA; 18 Adult Immunodeficiency Unit, Infectious Diseases, Inflammation Center, University of Helsinki and HUS Helsinki University Hospital, Helsinki, Finland; 19 Rare Disease Center and Pediatric Research Center, Children and Adolecents, University of Helsinki and HUS Helsinki University Hospital, Helsinki, Finland

**Keywords:** Toll-like receptor 3, hantavirus, central nervous system infections, encephalitis, primary immunodeficiency diseases, genetic diseases

## Abstract

Puumala hantavirus (PUUV) hemorrhagic fever with renal syndrome (HFRS) is common in Northern Europe; this infection is usually self-limited and severe complications are uncommon. PUUV and other hantaviruses, however, can rarely cause encephalitis. The pathogenesis of these rare and severe events is unknown. In this study, we explored the possibility that genetic defects in innate anti-viral immunity, as analogous to Toll-like receptor 3 (TLR3) mutations seen in HSV-1 encephalitis, may explain PUUV encephalitis. We completed exome sequencing of seven adult patients with encephalitis or encephalomyelitis during acute PUUV infection. We found heterozygosity for the TLR3 p.L742F novel variant in two of the seven unrelated patients (29%, *p* = 0.0195). TLR3-deficient P2.1 fibrosarcoma cell line and SV40-immortalized fibroblasts (SV40-fibroblasts) from patient skin expressing mutant or wild-type TLR3 were tested functionally. The TLR3 p.L742F allele displayed low poly(I:C)-stimulated cytokine induction when expressed in P2.1 cells. SV40-fibroblasts from three healthy controls produced increasing levels of IFN-λ and IL-6 after 24 h of stimulation with increasing concentrations of poly(I:C), whereas the production of the cytokines was impaired in TLR3 L742F/WT patient SV40-fibroblasts. Heterozygous TLR3 mutation may underlie not only HSV-1 encephalitis but also PUUV hantavirus encephalitis. Such possibility should be further explored in encephalitis caused by these and other hantaviruses.

**Electronic supplementary material:**

The online version of this article (10.1007/s10875-020-00834-2) contains supplementary material, which is available to authorized users.

## Introduction

Zoonotic RNA hantaviruses are carried and spread by rodents. The viruses are shed to rodent urine, droppings, and saliva, and they are mainly transmitted to human by inhalation. Hantaviruses may cause human disease with mortality [1]; hantavirus hemorrhagic fever with renal syndrome (HFRS) occurs in Europe and Asia whereas severe cardiopulmonary syndrome (HCPS) cases are seen in Americas [1, 2]. Puumala hantavirus (PUUV) HFRS is common in Europe with seroprevalence ranging from a few percent to approximately 13% in Finland [2]. Chronic or recurrent cases have not been reported. The elderly in rural environment especially in Northern Europe and those exposed to rodents are most commonly affected.

HFRS caused by PUUV primary infection is a complex acute febrile condition in which most symptoms arise from transient renal failure, disturbed tissue permeability, and tissue edema during febrile phase of the disease. Many PUUV HFRS patients also suffer from mostly secondary central nervous system (CNS) symptoms such as dizziness, headache, light sensitivity, and disturbed vision [3, 4]. Single cases of pituitary hemorrhage leading to panhypopituitarism during or soon after acute PUUV infection have been reported [5–7]. The patients may also rarely develop altered level of consciousness, personality change, new onset of focal neurological findings, or seizures consistent with encephalitis or acute encephalomyelitis [5, 8–10]. These unusual cases may also present with an elevated cerebrospinal fluid (CSF) white cell count, abnormal neuroimaging, or electroencephalography. PUUV hantavirus in the neuroendocrine cells and vascular endothelial cells of pituitary gland and in the CSF in single patients have been documented [5, 11]. PUUV IgM in the CSF suggestive of intrathecal antibody production has been found. In addition to PUUV HFRS, cases of Dobrava hantavirus HFRS encephalitis and Sin Nombre, Andes, and New York hantavirus HCPS encephalitis have been reported [12–15]. Young male patients seem to be at elevated risk of developing strong CNS symptoms associated with acute PUUV HFRS [16]. The pathogenesis of these rare and serious CNS events has remained unknown.

The aim of our study was to search for genetic explanation for encephalitis or encephalomyelitis caused by PUUV hantavirus. We enrolled all 7 PUUV HFRS patients hospitalized at Oulu University Hospital due to acute encephalitis or disseminated encephalomyelitis, diagnosed according to International Encephalitis Consortium diagnostic criteria [8]. We hypothesized that defective antiviral responses triggered by Toll-like receptor 3 (TLR3) can be responsible. TLR3 recognizes double-stranded RNA (dsRNA), an intermediate or by-product of replication by many viruses. Mutations in the *TLR3* gene predispose to herpes simplex virus 1 (HSV1) encephalitis (HSE), severe influenza pneumonia, and varicella zoster virus (VZV) ophthalmicus [17–19]. Limited evidence suggests that defective TLR3 signaling may also associate with a wider range of viral infections [20]. Our patients therefore underwent whole exome sequencing (WES) to test the hypothesis that their encephalitis may be associated with defective TLR3 signaling.

## Methods

### Inclusion Criteria

Adult patients with acute PUUV HFRS fulfilling the International Encephalitis Society criteria for encephalitis or encephalomyelitis were included [8]. Laboratory diagnosis of PUUV HFRS was based on serology analyzed using a commercial enzyme-linked immunosorbent assay of IgM antibodies (Reagena Puumala IgM EIA kit, Reagena, Toivala, Finland). In selected cases, the samples were also analyzed with indirect immunofluorescence test for PUUV IgG which displayed a granular staining pattern in cases of typical acute infection [2].

None of the patients (described in detail in Supplementary materials) with acute PUUV hantavirus HFRS suffered from a known primary or secondary immunodeficiency and tested negative for human immunodeficiency virus. They had symptoms and findings consistent with encephalitis (patients 2, 4, 5, 6) or acute disseminating encephalomyelitis (ADEM, patient 1). One patient developed aggressive multiorgan failure (patient 7). Patient 3 suffered from confusion, pituitary hemorrhage, and a prolonged ocular disease triggered by PUUV. Siblings of patient 1 were analyzed for TLR3 genetics and viral serology.

### Approval and Patient Consent

The study was conducted in accordance with principles of the Declaration of Helsinki and was approved by the Oulu University Hospital Ethics Committee. Written informed consent was obtained from the study subjects.

### Molecular Genetics

Genomic DNA was extracted from EDTA-blood samples using standard protocols. Briefly, the WES libraries were prepared according to manufacturer’s instructions at the Institute for Molecular Medicine Finland, Helsinki, Finland. In library preparation, the following kits were used: SureSelect Human All Exon V5 (Agilent Technologies, Santa Clara, CA, USA; patient 6), SureSelect Clinical Research Exome (Agilent Technologies, Santa Clara, CA, USA; patients 1, 5, and 7), Nextera Flex for Enrichment (Illumina, San Diego, CA, USA; patients 3 and 4), and SeqCap® EZ MedExome (Roche Diagnostics, Rotkreuz, Switzerland; patient 2). The sequencing was performed on HiSeq1500, HiSeq2500, or NovaSeq platforms (Illumina, San Diego, CA, USA). Sequencing reads were analyzed using in-house developed variant calling pipeline (VCP) for quality control, short read alignment, variant identification, and annotation [21]. Versions 3.1 (patient 6), 3.2 (patients 1, 5, and 7), and 3.7 (patients 2, 3, and 4) of VCP were used. Sanger sequencing was performed to confirm the *TLR3* variations, primer information and sequences are available upon a request.

The analysis included only exonic and splicing variants. Synonymous variants, variants with minor allele frequency (MAF) > 0.01 in Genome Aggregation Database (gnomAD; Cambridge, MA, USA; https://gnomad.broadinstitute.org/) and REVEL pathogenicity score < 0.3 were filtered out [22]. The analysis was targeted to known Primary Immune Deficiency Disease (PIDD) genes (in-house designed customized list of 513 genes) and to disease genes causative for encephalitis in Human Gene Mutation Database (HGMD; phenotype search “encephalitis,” list of 42 genes, July 2019). The remaining variants are listed in the Supplementary material Tables E1 (PIDD genes) and E2 (encephalitis genes); they were not validated by Sanger sequencing. In the variant prioritization in silico, prediction tools included in Annovar were utilized and the pathogenicity of variants was predicted according to the American College of Medical Genetics (ACMG) Standards and Guidelines [23–25]. Since the molecular genetic diagnosis remained unresolved, we loosened the filtering criteria for encephalitis disease genes: variants with MAF < 0.02 in Finnish populations listed in gnomAD (Cambridge, MA, USA; https://gnomad.broadinstitute.org/) and REVEL pathogenicity score > 0.2 were included [22, 24, 25].

### Cell Culture

The cell culture experiments to analyze the activity of the TLR3 p.L742F variant were conducted in generally accepted conditions as previously described [17, 18, 26]. Briefly, primary human fibroblasts were obtained from skin biopsies from patient 1 (P1) and healthy controls. The cells were transformed with SV40 vector to generate immortalized SV40-fibroblast cell lines as previously described [26]. The TLR3-deficient P2.1 fibrosarcoma cell line was provided by D.W. Leaman (University of Toledo, Toledo, OH). Stably transfected P2.1. cells were transfected with wild-type TLR3, or TLR3 L742F, R867Q or E746X mutants as previously described [18]. The SV40 fibroblasts and P2.1. cells were maintained in DMEM supplemented with 10% FCS.

TLR3 agonist poly(I:C) (Amersham) (concentrations 1, 5, and 25 μg/ml) was used as described [18]. The cells were stimulated with 25 μg/ml poly (I:C) with or without the presence of Lipofectamine (Invitrogen). Cells or supernatants were harvested, and their cytokine mRNA or protein production was analyzed by quantitative RT-PCR or ELISA as described [18].

### HSV-1 and HSV-2 Serology

HSV-1 and HSV-2 type specific IgG antibodies were performed using HerpeSelect (Focus Diagnostics) ELISA kit according to the manufacturer’s instructions at the Department of Medical Microbiology, Turku University Hospital, Finland. The index values < 0.9 were defined as negative, 0.9–1.10 as a borderline and > 1.10 as positive result.

## Results

### Genetic Analysis

**Fig. 1:**
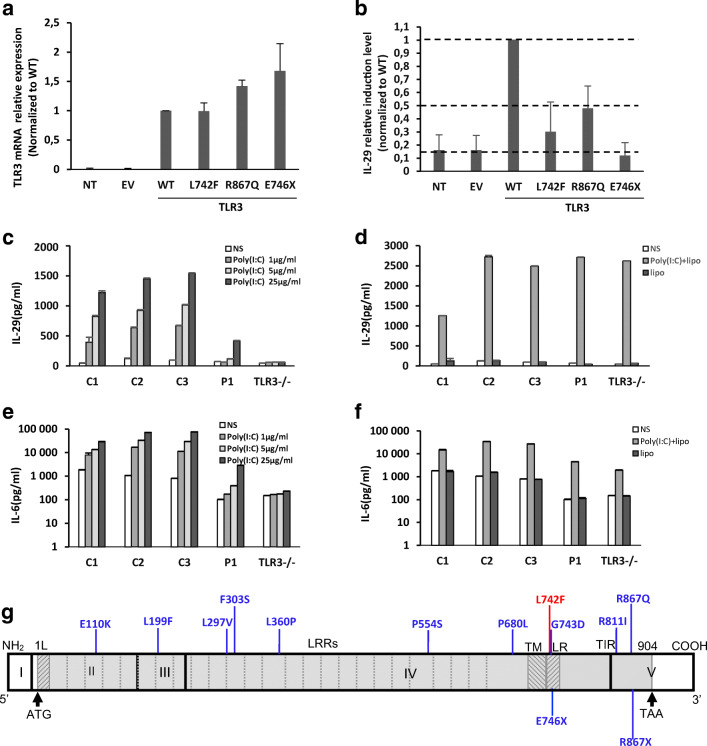
TLR3 mRNA expression levels were determined by RT-qPCR, as normalized to wild-type (WT) relative expression levels, in P2.1 TLR3-deficient fibrosarcoma cells without transfection (NT) or transfected with empty vector (EV), HA-tagged TLR3 WT, L742F (p.Leu742Phe), R867Q (p.Arg867Gln), or E746X (**a**). *IFNL1 (IL29)* induction by poly(I:C) stimulation, as normalized to wild-type (WT) fold induction level, in P2.1 cells not transfected (P2.1) or stably transfected with empty vector (P2.1+EV), HA-tagged *TLR3* WT, L742F, R867Q, or E746X (**b**). Production of IL-29 (**c** and **d**), and IL-6 (**e** and **f**) in SV40-fibroblasts from three healthy controls (C1, C2, C3), P1, and a TLR3−/− HSE patient, 24 h after stimulation with 1, 5, or 25 μg/ml poly(I:C) (**c** and **e**), or with 25 μg/ml poly(I:C) in the presence of lipofectamine (poly(I:C)+lipo; **d** and **f**), or lipofectamine alone, as assessed by ELISA. Schematic structure of the human *TLR3* gene and protein, featuring the leader sequence (L), leucine-rich repeats (LRRs) of the ectodomain, transmembrane domain (TM), linker region (LR), and Toll/IL-1 receptor (TIR) domain. Roman numerals indicate the coding exons. Previously reported mutations found in patients with HSE patients (E110K, L297V, L360P, P554S, G743D, R811I, R867Q), severe influenza (F303S, P554S, P680L) or Varicella zoster virus infection (L199F, R867X), that have been previously experimentally characterized (F303S, L360P, P554S, P680L, G743D, R811I, R867Q, R867X) or not (E110K, L199F, L297V) are shown in blue. The L742F mutation found in the two patients with complicated Puumala hantavirus infection is shown in red (**g**)

Exome sequencing identified patients 1 and 7 to carry the same heterozygous TLR3 variation (rs147431766; chr4:187005064C>T ENSG00000164342:ENST00000296795:exon4:c.C2224T:p.L742F) which is enriched in the Finnish population (allele frequency (AF) 0.01621 in Finnish population) compared to more heterogenous European population (AF 0.0007 in European non-Finnish, Genome Aggregation Database; gnomAD; https://gnomad.broadinstitute.org/), and has not been described in any human patients. Regardless, the p.L742F variation is significantly enriched in our patient cohort (29%, *p* = 0.0195) compared to general Finnish population. Other two patients, patients 2 and 3, are heterozygous for a common TLR3 p.L412F variation (rs3775291, AF 0.324487 in Finnish population), but this variant showed no enrichment in our patients. The TLR3 variants were validated by Sanger sequencing in all patients. The L742F variant found in our PUUV HFRS patients is shown in Fig. 1g. Figure 1 g also illustrates previously characterized TLR3 mutations associated with HSE, severe influenza, and other viral infections [17–20].

We performed family segregation of the p.L742F TLR3 variation, using DNA from siblings of patient 1. We found that a total of 6 of the 9 analyzed family members were positive for the TLR3 p.L742F variant (Supplementary material Figure, family tree of patient 1). Patient 7 (p.L742F) did not have siblings, he lived with his grandmother, and his parents were not available for analysis. Family members of patients 2 and 3, who are heterozygous for the TLR3 p.L412F common variant, were not tested, as this variation is not enriched in our patients.

In addition to TLR3 variants, several genetic findings of uncertain significance were identified in the whole exome sequencing data of patients 1 and 7 (Supplementary material Tables E1, E2, and E3). Interestingly, patient 7 carried a heterozygous myosin-binding protein C mutation (chr11:47354442C>T, c.G3413A, p.R1138H, rs187705120) known to cause hypertrophic cardiomyopathy (OMIM 600958), which may have contributed to his severe phenotype.

### The L742F TLR3 Protein Is Severely Hypomorphic In Vitro

We aimed to analyze the effect of the TLR3 L742F variant in the TLR3-deficient P2.1 fibrosarcoma cell line, which does not produce detectable amounts of TLR3 protein and does not respond to the dsRNA mimic polyinosinic:polycytidylic acid (poly[I:C]) [32]. To do so, we generated cell lines stably transfected with empty plasmid or with plasmids containing C-terminally HA-tagged WT and mutants TLR3 cDNAs. In P2.1 cells expressing WT TLR3, the production of *IFNL1* mRNAs was induced after poly(I:C) stimulation, whereas cells expressing the L742F displayed low poly(I:C)-stimulated induction of *IFNL1* mRNA, like those expressing the previously reported R867Q allele, while the previously reported E746X allele displayed no induction *of IFNL1* (E746X) (Fig. 1a, b). We chose not to analyze activity of the TLR3 p.L412F variation, as this variant is not enriched in our patients and it is thoroughly analyzed by previously studies in different conditions. [27–31]

### Impaired Responses to Poly(I:C) in Patient Fibroblasts Heterozygous for TLR3 L742F

We further tested whether heterozygosity for the *TLR3* L742F mutation is related to an AD TLR3 deficiency at the cellular level. Human dermal fibroblasts respond to extracellular poly(I:C) stimulation in a TLR3-dependent manner [17, 26, 33]. We studied the response to poly(I:C) in SV40-immortalized skin fibroblasts (SV40-fibroblasts) from P1 (L742F/WT), three healthy individuals and a HSE patient with AR complete TLR3 deficiency (TLR3^−/−^, due to compound heterozygous P554S and E746X mutations) [26, 33] (Fig. 1c–f). The fibroblasts from the three healthy controls produced increasing levels of IFN-λ and IL-6 after 24 h of stimulation with increasing concentrations of poly(I:C), whereas the production of the cytokines was impaired in P1 TLR3 L742F/WT and abolished in TLR3^−/−^ fibroblasts.

### HSV and PUUV Serology Study in Patient Families

We 9 family members of patient 1 for PUUV hantavirus, HSV1 and HSV2 serology (Supplementary material Figure, Family 1). All tested family members were positive for HSV1 and negative for HSV2 antibodies. In addition to the index (P1), only one TLR3 heterozygous sibling had PUUV hantavirus IgG. He was also HSV-1 seropositive. None of the family members had suffered from an episode of encephalitis. Incomplete penetrance for HSE is well documented in families with heterozygous TLR3 variations, including in individuals infected with HSV-1, as is expected for sporadic encephalitis [17, 26]. The patients and their family members were not analyzed for influenza virus or VZV antibodies.

## Discussion

Inborn errors in TLR3-mediated immune response can explain at least severe HSV1 and influenza complications in some patients [18, 26]. Our current study suggests that TLR3 deficiency may predispose also to hantavirus encephalitis. In previously described HSV1 and influenza cases, poor TLR3-mediated in vitro interferon production has been documented. In agreement with these previous results, heterozygosity of the TLR3 p.L742F mutation was related to significantly compromised responses to TLR3 stimulation in patient fibroblasts, in terms of interferon and IL-6 induction. The TLR3 activities were examined in widely used cell culture conditions including a TLR3 deficient cell line and SV40 immortalized patient skin fibroblasts. Our results are also supported by the previously published data on ability of hantaviruses to activate TLR3-dependent immune responses in vitro [34, 35].

Hantavirus HFRS is a complex acute condition during which a sequence of diverse presentations in most organs of human body can be observed. PUUV HFRS patients experience, for example, febrile phase with acute kidney failure, and they have disturbed tissue permeability with edema. This is followed by the recovery of kidney function during which the urine output can be extremely high. Most patients suffer from mild CNS symptoms such as head ache and dizziness during this dynamic disease development. A very low number of these HFRS patients, however, develop obvious symptomatic encephalitis that fulfills the accepted criteria [8]. When compared to HSE, for example, the CNS symptoms in PUUV HRFS encephalitis are obviously more diverse. This variability in the PUUV HFRS encephalitis presentation is well demonstrated in our patient cases described in detail in Supplementary materials.

Exome sequencing can identify known monogenic disease-causing genes in approximately 10 to 20% of patients with primary immunodeficiency [36]. In our study, the TLR3 p.L742F variant showed reduced biological activity, and significant enrichment in our cohort of patients as it was found in two of the seven cases. When compared with HSE patients, TLR3 deficiency was found in 6 (5%) of the 120 HSE cases reported [17]. We found the TLR3 p.L742F variant to be significantly more common in our patient cohort compared to general Finnish population (29%, *p* = 0.0195). Although the role of TLR3 p.L742F appears important, we cannot exclude the possibility that other genes may also contribute to the described events. For example, the heterozygous myosin-binding protein C mutation in patient 7 may have affected the course of his disease. Also, consequences of this mutation on immune responses in primary human PUUV infection should require further studies [37]. The TLR3 p.L412F variant without enrichment in our patient population was observed in another two patients; this common variant exhibit neutral functional testing and it is not associated with any disease condition in heterozygous form [27–30]. All cases and their family members with the hypormophic TLR3 p.L742F variant or the common p.L412F variant were positive for HSV1 antibodies; none of them had a history of HSE. One patient with p.L412F developed chronic eye symptoms soon after the acute PUUV infection (patient 3, Supplementary material). Ocular features are among the most common presentations of PUUV hantavirus infection, although this may be not related to the TLR3 p.L412F variation at all [2].

Several hantavirus species can cause encephalitis of varying severity in humans. Our current study may explain for the first time genetic and biological mechanisms for these most severe CNS conditions caused by the hantaviruses. It seems possible that genetic defects in antiviral response should at least partly explain these potentially life-threatening conditions. PUUV HFRS in our study represents a mild form of hantavirus disease. It is possible that encephalitis caused by other hantaviruses with a higher rate of complications and mortality can be explained with similar genetic and biological mechanisms. We recommend further studies to explore the potential of defective TLR3 signaling or other innate antiviral responses to associate with severe complications upon infections with other hantavirus species [26, 33].

## Electronic supplementary material

ESM 1 (XLSX 14 kb)

ESM 2 (XLSX 10 kb)

ESM 3 (XLSX 15 kb)

ESM 4 (DOCX 97 kb)

## Abbreviations

HFRS: Hemorrhagic fever with renal syndrome
HCPS: Hantavirus cardiopulmonary syndrome
PUUV: Puumala hantavirus
TLR3: Toll-like receptor 3
HSV: Herpes simplex virus
HSE: Herpes simplex virus encephalitis
WES: Whole exome sequencing
ADEM: Acute disseminating encephalitis

## References

[1] Avsic-Zupanc T, Saksida A, Korva M (2019). Hantavirus infections. Clin Microbiol Infect.

[2] Vaheri A, Henttonen H, Voutilainen L, Mustonen J, Sironen T, Vapalahti O (2013). Hantavirus infections in Europe and their impact on public health. Rev Med Virol.

[3] Hautala T, Mähonen SM, Sironen T, Hautala N, Pääkkö E, Karttunen A (2010). Central nervous system-related symptoms and findings are common in acute Puumala hantavirus infection. Ann Med.

[4] Alexeyev OA, Morozov VG (1995). Neurological manifestations of hemorrhagic fever with renal syndrome caused by Puumala virus: review of 811 cases. Clin Infect Dis.

[5] Hautala T, Sironen T, Vapalahti O, Pääkko E, Särkioja T, Salmela PI (2002). Hypophyseal hemorrhage and panhypopituitarism during Puumala virus infection: magnetic resonance imaging and detection of viral antigen in the hypophysis. Clin Infect Dis.

[6] Pekic S, Cvijovic G, Stojanovic M, Kendereski A, Micic D, Popovic V (2005). Hypopituitarism as a late complication of hemorrhagic fever. Endocrine..

[7] Stojanovic M, Pekic S, Cvijovic G, Miljic D, Doknic M, Nikolic-Djurovic M, Micic D, Hrvacevic R, Nesic V, Popovic V (2008). High risk of hypopituitarism in patients who recovered from hemorrhagic fever with renal syndrome. J Clin Endocrinol Metab.

[8] Venkatesan A, Tunkel AR, Bloch KC, Lauring AS, Sejvar J, Bitnun A, Stahl JP, Mailles A, Drebot M, Rupprecht CE, Yoder J, Cope JR, Wilson MR, Whitley RJ, Sullivan J, Granerod J, Jones C, Eastwood K, Ward KN, Durrheim DN, Solbrig MV, Guo-Dong L, Glaser CA, Sheriff H, Brown D, Farnon E, Messenger S, Paterson B, Soldatos A, Roy S, Visvesvara G, Beach M, Nasci R, Pertowski C, Schmid S, Rascoe L, Montgomery J, Tong S, Breiman R, Franka R, Keuhnert M, Angulo F, Cherry J, on behalf of the International Encephalitis Consortium (2013). Case definitions, diagnostic algorithms, and priorities in encephalitis: consensus statement of the international encephalitis consortium. Clin Infect Dis.

[9] Bergmann F, Krone B, Bleich S, Prange H, Paulus W (2002). Encephalitis due to a hantavirus infection. J Inf Secur.

[10] Krause R, Aberle S, Haberl R, Daxbock F, Wenisch C (2003). Puumala virus infection with acute disseminated encephalomyelitis and multiorgan failure. Emerg Infect Dis.

[11] Mähönen SM, Sironen T, Vapalahti O, Pääkkö E, Hautala N, Ilonen J, Glumoff V, Vainio O, Kauma H, Vaheri A, Plyusnin A, Hautala T (2007). Puumala virus RNA in cerebrospinal fluid in a patient with uncomplicated nephropathia epidemica. J Clin Virol.

[12] Cerar D, vsic-Zupanc T, Jereb M, Strle F (2007). Case report: severe neurological manifestation of Dobrava hantavirus infection. J Med Virol..

[13] Huisa BN, Chapin JE, Adair JC (2009). Central nervous system complications following Hanta virus cardiopulmonary syndrome. J Neuro-Oncol.

[14] Talamonti L, Padula PJ, Canteli MS, Posner F, Marczeski FP, Weller C (2011). Hantavirus pulmonary syndrome: encephalitis caused by virus Andes. J Neuro-Oncol.

[15] Fernando R, Capone D, Elrich S, Mantovani R, Quarles L, D'Amato A (2019). Infection with New York orthohantavirus and associated respiratory failure and multiple cerebral complications. Emerg Infect Dis.

[16] Hautala T, Hautala N, Mähonen SM, Sironen T, Pääkko E, Karttunen A (2011). Young male patients are at elevated risk of developing serious central nervous system complications during acute Puumala hantavirus infection. BMC Infect Dis.

[17] Lim HK, Seppänen M, Hautala T, Ciancanelli MJ, Itan Y, Lafaille FG (2014). TLR3 deficiency in herpes simplex encephalitis: high allelic heterogeneity and recurrence risk. Neurology..

[18] Lim HK, Huang SXL, Chen J, Kerner G, Gilliaux O, Bastard P, Dobbs K, Hernandez N, Goudin N, Hasek ML, García Reino EJ, Lafaille FG, Lorenzo L, Luthra P, Kochetkov T, Bigio B, Boucherit S, Rozenberg F, Vedrinne C, Keller MD, Itan Y, García-Sastre A, Celard M, Orange JS, Ciancanelli MJ, Meyts I, Zhang Q, Abel L, Notarangelo LD, Snoeck HW, Casanova JL, Zhang SY (2019). Severe influenza pneumonitis in children with inherited TLR3 deficiency. J Exp Med.

[19] Sironi M, Peri AM, Cagliani R, Forni D, Riva S, Biasin M, Clerici M, Gori A (2017). TLR3 mutations in adult patients with herpes simplex virus and varicella-zoster virus encephalitis. J Infect Dis.

[20] Zhang SY, Herman M, Ciancanelli MJ, de Perez DR, Sancho-Shimizu V, Abel L (2013). TLR3 immunity to infection in mice and humans. Curr Opin Immunol.

[21] Sulonen AM, Ellonen P, Almusa H, Lepistö M, Eldfors S, Hannula S (2011). Comparison of solution-based exome capture methods for next generation sequencing. Genome Biol.

[22] Ioannidis NM, Rothstein JH, Pejaver V, Middha S, McDonnell SK, Baheti S (2016). REVEL: an ensemble method for predicting the pathogenicity of rare missense variants. Am J Hum Genet.

[23] Li Q, Wang K (2017). InterVar: clinical interpretation of genetic variants by the 2015 ACMG-AMP guidelines. Am J Hum Genet.

[24] Rentzsch P, Witten D, Cooper GM, Shendure J, Kircher M (2019). CADD: predicting the deleteriousness of variants throughout the human genome. Nucleic Acids Res.

[25] Davydov EV, Goode DL, Sirota M, Cooper GM, Sidow A, Batzoglou S (2010). Identifying a high fraction of the human genome to be under selective constraint using GERP++. PLoS Comput Biol.

[26] Zhang SY, Jouanguy E, Ugolini S, Smahi A, Elain G, Romero P, Segal D, Sancho-Shimizu V, Lorenzo L, Puel A, Picard C, Chapgier A, Plancoulaine S, Titeux M, Cognet C, von Bernuth H, Ku CL, Casrouge A, Zhang XX, Barreiro L, Leonard J, Hamilton C, Lebon P, Heron B, Vallee L, Quintana-Murci L, Hovnanian A, Rozenberg F, Vivier E, Geissmann F, Tardieu M, Abel L, Casanova JL (2007). TLR3 deficiency in patients with herpes simplex encephalitis. Science..

[27] Ranjith-Kumar CT, Miller W, Sun J, Xiong J, Santos J, Yarbrough I, Lamb RJ, Mills J, Duffy KE, Hoose S, Cunningham M, Holzenburg A, Mbow ML, Sarisky RT, Kao CC (2007). Effects of single nucleotide polymorphisms on Toll-like receptor 3 activity and expression in cultured cells. J Biol Chem.

[28] Willmann O, Ahmad-Nejad P, Neumaier M, Hennerici MG, Fatar M (2010). Toll-like receptor 3 immune deficiency may be causative for HSV-2-associated mollaret meningitis. Eur Neurol.

[29] Yang CA, Raftery MJ, Hamann L, Guerreiro M, Grutz G, Haase D (2012). Association of TLR3-hyporesponsiveness and functional TLR3 L412F polymorphism with recurrent herpes labialis. Hum Immunol.

[30] Cooke G, Kamal I, Strengert M, Hams E, Mawhinney L, Tynan A, O’Reilly C, O’Dwyer DN, Kunkel SL, Knaus UG, Shields DC, Moller DR, Bowie AG, Fallon PG, Hogaboam CM, Armstrong ME, Donnelly SC (2018). Toll-like receptor 3 L412F polymorphism promotes a persistent clinical phenotype in pulmonary sarcoidosis. QJM..

[31] Gosu V, Son S, Shin D, Song KD (2019). Insights into the dynamic nature of the dsRNA-bound TLR3 complex. Sci Rep.

[32] Sun Y, Leaman DW (2004). Ectopic expression of toll-like receptor-3 (TLR-3) overcomes the double-stranded RNA (dsRNA) signaling defects of P2.1 cells. J Interferon Cytokine Res.

[33] Guo Y, Audry M, Ciancanelli M, Alsina L, Azevedo J, Herman M, Anguiano E, Sancho-Shimizu V, Lorenzo L, Pauwels E, Philippe PB, Pérez de Diego R, Cardon A, Vogt G, Picard C, Andrianirina ZZ, Rozenberg F, Lebon P, Plancoulaine S, Tardieu M, Doireau V, Jouanguy E, Chaussabel D, Geissmann F, Abel L, Casanova JL, Zhang SY (2011). Herpes simplex virus encephalitis in a patient with complete TLR3 deficiency: TLR3 is otherwise redundant in protective immunity. J Exp Med.

[34] Handke W, Oelschlegel R, Franke R, Kruger DH (2009). Rang A Hantaan virus triggers TLR3-dependent innate immune responses. J Immunol.

[35] Zhang Y, Liu B, Ma Y, Yi J, Zhang C, Zhang Y (2014). Hantaan virus infection induces CXCL10 expression through TLR3, RIG-I, and MDA-5 pathways correlated with the disease severity. Mediat Inflamm.

[36] Meyts I, Bosch B, Bolze A, Boisson B, Itan Y, Belkadi A, Pedergnana V, Moens L, Picard C, Cobat A, Bossuyt X, Abel L, Casanova JL (2016). Exome and genome sequencing for inborn errors of immunity. J Allergy Clin Immunol.

[37] Resman RK, Korva M, Bogovic P, Pal E, Strle F, Avsic-Zupanc T (2018). Delayed interferon type 1-induced antiviral state is a potential factor for hemorrhagic fever with renal syndrome severity. J Infect Dis.

