# Supplementary material for: Heterozygous TLR3 Mutation in Patients with Hantavirus Encephalitis
Source: J Clin Immunol. 2020 Sep 16;40(8):1156–62. doi: 10.1007/s10875-020-00834-2 (PMC7567724; doi:10.1007/s10875-020-00834-2)
Supplement: Supplementary file 4 — (DOCX 97 kb) [file 10875_2020_834_MOESM4_ESM.docx]

**Supplementary material to**

**Heterozygous TLR3 mutations in patients with hantavirus encephalitis**

Terhi Partanen^1^, Jie Chen^2^, Johanna Lehtonen^11,12^, Outi Kuismin^3^, Harri Rusanen^4^, Olli Vapalahti^5^, Antti Vaheri^5^,Veli-Jukka Anttila^6^, Michaela Bode^7^, Nina Hautala^8^, Tytti Vuorinen^9^, Virpi Glumoff^10^,Minna Kraatari^3^, Pirjo Åström^10^, Janna Saarela^11,13^, Heikki Kauma^1^,

Lazaro Lorenzo^14^, Jean-Laurent Casanova^2,14,15,16,17^, Shen-Ying Zhang^2,14,15^,

Mikko Seppänen^18,19^, Timo Hautala^1,10^

**CASE DESCRIPTIONS**

All patient cases included in this study were confirmed to have acute Puumala hantavirus (PUUV) hemorrhagic fever with renal syndrome (HFRS) based on anti-PUUV IgM titers. They presented with a typical multi-organ dynamic disease process of transient kidney failure, permeability disorder and tissue edema. In addition to typical HFRS features, they also suffered an episode of encephalitis or acute disseminated encephalomyelitis during or soon after their HFRS. The neurological symptoms and findings are consistent with the widely accepted encephalitis criteria.[1]

**Patient 1 (P1)**

Patient 1 is a 65-year-old male with history of prostate hyperplasia. The patient and his family originate from a sparsely populated northeastern part of Finland; genetically, his parents may have been related.

Before his contact with health care, he first developed flu-like symptoms, constipation and abdominal tenderness associated with nausea and vomiting for 5 days. At the first hospital contact, he complained epigastric pain. Plain abdominal x-ray and abdominal ultrasound revealed small amounts of ascites and his kidneys appeared hyperechogenic. Chest x-ray showed mild pulmonary hyperemia with moderate pleural effusion. His blood pressure was 120/85 mmHg and his respiratory rate was normal. Blood white cell count was 18.3 × 10^9^/l (normal range 3.4-8.2 × 10^9^/l) and his blood thrombocyte count was low 36 × 10^9^/l (normal range 150-360 × 10^9^/l). His plasma creatinine was elevated. Urine sample was positive for erythrocytes and albumin. His clinical condition was suggestive of acute Puumala hantavirus (PUUV) hemorrhagic fever with renal syndrome (HFRS). This was confirmed with PUUV IgM enzyme immunoassay (EIA) based on recombinant nucleocapsid antigen. The result was strongly positive (absorbance value 159, cut-off 30) and consistent with the diagnosis of acute PUUV HFRS.

The patient was hospitalized and developed oliguria as a typical HFRS clinical presentation. The lowest daily urine output during the oliguric phase of the disease was 200 ml with the peak plasma creatinine concentration of 736 µmol/l (normal range 60 – 100 µmol/l). His body weight increased to 11 kg above normal due to fluid retention and peripheral edema, with markedly elevated blood pressure. Fluid retention, urine output and plasma creatinine concentration were improving already on the 6^th^ day of hospitalization.

On the 10^th^ day of hospitalization, he developed headache and confusion, and was unable to follow simple instructions. Neurological examination revealed sensitization to touch and feeling to pinching below neck level, as well as reflection sensitization on lower extremities. Clinical presentation was consistent with acute disseminated encephalomyelitis (ADEM).

Cerebrospinal fluid (CSF) was sampled due to acute neurological symptoms. CSF white cell count (11 × 10^6^/l; normal range <3) and protein concentration were elevated (701 mg/l, normal range 150-500). CSF was negative for bacteria and displayed no oligoclonality. CSF PUUV IgM, IgG, and nucleic acid testing remained negative. CSF was also negative for herpes simplex virus (HSV) 1 and 2, varicella zoster virus (VZV), enterovirus and parechovirus nucleic acids. *Borrelia* sp., *Mycoplasma pneumoniae*, N-methyl-D-aspartate receptor (NMDAR) and voltage-dependent potassium channel (anti-VGKC) antibodies were negative. Brain magnetic resonance imaging (MRI) demonstrated multiple supratentorial white matter lesions, with frontoparietal predilection, possibly of vascular origin. In addition, T2-hyperintense lesions were found in the cerebellum and possibly represented acute inflammatory changes.

He received methylprednisolone 1000 mg/day intravenously on three consecutive days, directed at ADEM associated with PUUV HFRS. This lead to rapid improvement of his clinical condition; he achieved full mental and physical recovery.

Brain MRI was repeated at one and four months: the supratentorial white matter lesions remained unchanged, but cerebellar pathology had resolved. Spinal MRI showed no evidence of myelitis or other spinal cord lesions. Magnetic resonance angiography at four months showed no significant stenosis of the carotid or vertebral arteries.

**Patient 2 (P2)**

A 64-year old male with a history of hypertension developed acute high fever. One day later he developed confusion, fluctuating consciousness and myoclonic movements. He also developed oliguria and respiratory distress, was admitted to intensive care unit and required mechanical ventilation for two days. He received intravenous catecholamine infusions and empiric antimicrobial treatment with meropenem and acyclovir. His myoclonic movements were controlled with intravenous lorazepam.

Electroencephalography (EEG) was abnormal with generalized non-specific slowing. Brain computed tomography (CT) was normal. At admission to intensive care, his chest X-ray was unremarkable, but later he developed pleural effusion.

His plasma creatinine was elevated (307 µmol/l, normal range 60-100 µmol/l), blood white cell count was high (15.7 × 10E9/l, normal range 3.4-8.2 × 10^9^/l) with mild thrombocytopenia (127 x 10^9^/l, 150-360 x 10^9^/l), and his C-reactive protein was 65 mg/l (normal <10 mg/l). Tests for respiratory viruses and blood cultures remained negative. Positive serum PUUV IgM confirmed PUUV HFRS diagnosis. CSF showed mildly elevated white cell count (5 x 10^6^/l, normal <3 x 10^6^/l) and CSF protein concentration (1114 mg/l, normal range 150-500 mg/l). CSF tested negative for HSV1 and 2 nucleic acids.

After two days of mechanical ventilation, he underwent quick recovery. Although he continued to suffer from high fevers, his kidney function, consciousness and neurological condition started improving on the third day of hospitalization. After two weeks of hospitalization, his neurological condition was considered normal.

**Patient 3 (P3)**

A 49-year old, previously healthy male developed acute high fever. On the fifth day of his illness he experienced oliguria, vomiting and transient vison loss lasting for minutes. At hospital admission, he suffered from high fever, intensive headache, confusion and somnolence.

His thrombocyte count was low (4 x 10^9^/l, normal range 150-360 x 10^9^/l) and plasma creatinine elevated (411 µmol/l, normal range 60-100 µmol/l). Urine sample was positive for erythrocytes. PUUV IgM tested positive. CSF sample was collected on the11^th^ day of fever, when thrombocytopenia had resolved; CSF white cell count was 5 x 10^6^/l (normal <3 x 10^6^/l) and protein concentration 1155 mg/l (normal range 150-500 mg/l).

Because of headache, confusion and somnolence, brain computed tomography (CT) was performed with normal findings. Brain MRI, however, revealed pituitary hemorrhage. EEG showed mild left temporal non-specific disturbances.

After acute hospitalization, the patient developed sustained panhypopituitarism with a need for hormonal replacement therapy. He continued to suffer from severe chronic neuralgia on the left side of head, dizziness and fatigue, leading to disability and retirement. Simultaneously with headache attacks, he also continued to suffer from recurrent episodes of blurred visual acuity, increased lacrimation and upper lid ptosis in his left eye. These symptoms lasted for 30 to 120 minutes and they reoccurred several years after the acute HFRS episode.

**Patient 4 (P4)**

A 47-year-old previously healthy female developed acute nausea, vomiting, headache, photophobia and high fever. She also developed petechiae, hypotension and anuria.

Soon after hospital admission, she developed circulatory insufficiency (blood pressure 65/49) and metabolic acidosis necessitating intensive care, fluid resuscitation and intravenous catecholamines. On the 6^th^ day of illness, her consciousness continued to deteriorate, with confusion and somnolence. She received empiric acyclovir and ceftriaxone.

Acute phase testing showed thrombocytopenia (9 x 10^9^/l, normal range 150-360 x 10^9^/l), elevated plasma creatinine (176 µmol/l, normal range 60-100 µmol/l), and her urine was positive for erythrocytes and albumin. She was positive for PUUV IgM, confirming PUUV HFRS.

Brain CT scan was normal. Brain MRI was consistent with pituitary hemorrhage. EEG showed unspecific abnormal occipital activity without epileptiform features.

Her CSF was taken by lumbar puncture after thrombocytopenia had resolved, on the 10^th^ day of fever: mild elevation in white cell count (18 x 10^6^/l, normal < 3 x 10^6^/l) and elevated protein concentration (1527 mg/l, normal range 150-500mg/l) were found. CSF tested negative for HSV and VZV nucleic acids.

After 12 days of hospital care, she was released in good condition. At follow up, she developed panhypopituitarism; sustained hormonal replacement therapy was needed.

**Patient 5 (P5)**

After the onset of acute headache, dizziness, oliguria, nausea and high fever, a 17-year-old male known to suffer from mild allergic asthma was hospitalized, on the 7^th^ day of illness. He suffered from headache, altered consciousness and tonic-clonic convulsions requiring intensive care. He received repeated doses of intravenous lorazepam and phenytoin treatment to control the seizures. He also received empiric acyclovir and ceftriaxone.

Laboratory tests showed thrombocytopenia 62 x 10E9/l (normal range 150-360 x 10E9/l), elevated creatinine 230 µmol/l (60-100 µmol/l), hematuria and albuminuria. PUUV IgM tested positive. CSF white cell count was 5 x 10^6^/l (normal < 3 x 10^6^/l), protein concentration was 448 mg/l (normal range 150-500mg/l) and the CSF HSV and VZV nucleic acid analyses were negative.

EEG analyzed during the antiepileptic medication was negative for epileptiform irritation and brain CT was unremarkable. Brain MRI showed temporo-occipital cortical edema extending to the parietal and frontal regions.

The epileptic seizures resolved after three days of hospitalization. The high fever and kidney failure were also improving after four days. The patient finally made a full neurological and physical recovery.

**Patient 6 (P6)**

A 19-year-old male, known to suffer from asthma, developed flu-like symptoms and received cephalexin to treat upper respiratory tract infection. Soon after, he developed vomiting, weakness and deterioration of consciousness.

At hospital admission, he had thrombocytopenia (13 x 10^9^/l, normal range 150-360 x 10^9^/l), elevated plasma creatinine concentration (167 µmol/l, normal range 60-100 µmol/l), hematuria and albuminuria. PUUV IgM was positive consistent with the PUUV HFRS diagnosis.

The patient developed pulmonary edema and respiratory failure. He required mechanical ventilation for three days. EEG presented with generalized slowing disorder and focal encephalitis in the left temporal region. Brain CT scan was normal and brain MRI showed hypophyseal hemorrhage.

CSF white cell count (420 x 10^6^/l, normal < 3 x 10E6/l) and protein concentration (2314 ml/l, normal range 150-500mg/l) were elevated. Blood and CSF bacterial and viral analyses, including HSV nucleic acids were negative.

Control CSF sample continued to be positive for white cells (9 x 10^6^/l, normal < 3 x 10^6^/l), elevated protein concentration (715 ml/l, normal range 150-500mg/l) and erythrocytes (457 x 10^6^/l, normal range 0-1 x 10^6^/l). Empiric wide spectrum antimicrobials (meropenem, vancomycin, acyclovir) were given though CSF search for pathogens remained negative.

He developed panhypopituitarism and hormonal replacement therapy was given. On the 14^th^ day, repeated brain MRI showed that the pituitary hemorrhage had partly resolved. He was released from the hospital in good condition after 17 days of hospitalization. One year after the acute episode, he had fully recovered from hypopituitarism.

**Patient 7 (P7)**

Patient 7 was a 19-year-old previously healthy male. He lived with his grandmother and had no siblings. His parents were not available for genetic analysis.

He had suffered from high fever for three days before hospital admission and noticed low urine output and loss of thirst. At admission, he had high fever of 39.8$^{\circ} C$ and appeared dehydrated. Soon after hospitalization, he suffered from altered consciousness followed by respiratory distress. He received enhanced intravenous fluids, his fluid balance became positive and oxygen saturation fell to 91% despite receiving 2 l/min of oxygen through nasal cannulation. Mild pulmonary edema was detected in chest X-ray. Abdominal ultrasound revealed vena portae occlusion. The patient was admitted to intensive care unit (ICU) because of developing septic shock and multiorgan failure.

He developed clinical signs suggesting acute abdominal complications. He underwent laparotomy due to high intra-abdominal pressure (IAP 22 mmHg), with vacuum-assisted closure. He also suffered from left ventricular failure with left ventricular ejection fraction of only 15-20%. Septic shock was not controlled despite vasoactive drugs and broad-spectrum antibiotics. Oxygenation failure necessitated extracorporeal membrane oxygenation (ECMO, femoral veno-arterious). Despite all attempts, he died of multiorgan failure.

At hospital admission, blood hemoglobin was 144 g/l and blood white cell count was 3.7 x10^9^/l, with lymphopenia (0.58 x10^9^/l; normal range 1.3-3.6 x10^9^/l), normal neutrophil level 3.58 x10^9^/l and thrombocytopenia (55 x10^9^/l, normal range 150-360 x10^9^/l). C-reactive protein (CRP) level was 94 mg/l (normal <3 mg/l), plasma creatinine 112 µmol/l (normal range 60-100 µmol/l), potassium and sodium concentrations were 3,4 mmol/l (normal range 3.5-4.8 mmol/l) and 135 mmol/l (normal range 137-145 mmol/l), respectively. Serum alanine aminotransferase level was elevated 604 U/L (normal range <50 U/l). Urine erythrocyte 30x10^6^/l (normal range < 20^6^/l) and leukocyte (89 x10^6^, normal range <10 x10^6^/l) counts were increased; blood and urine bacterial cultures were negative and chest X-ray was normal. PUUV IgM antibodies were positive consistent with PUUV HFRS.

Post-mortem examination was performed. Histology of the brain revealed mild ischemic lesions, but no evidence of encephalitis. The kidneys displayed findings consistent with PUUV HFRS, with autolytic tubular epithelium and in medullae, with marked hemorrhage. Congestion and pericentral necrosis of the liver were detected. Findings of hypertrophic cardiomyopathy were also noticed. Heart was firm and spherical in shape. Left ventricular wall was hypertrophic, lumen of left ventricle was narrow and papillary muscles were hypertrophic. Microscopic examination of the heart showed narrow fibrotic tracts in patches and a mild disorder of monocytes. Some CD3 positive T-lymphocytes were found, but findings to suggest lymphocytic myocarditis were not seen.


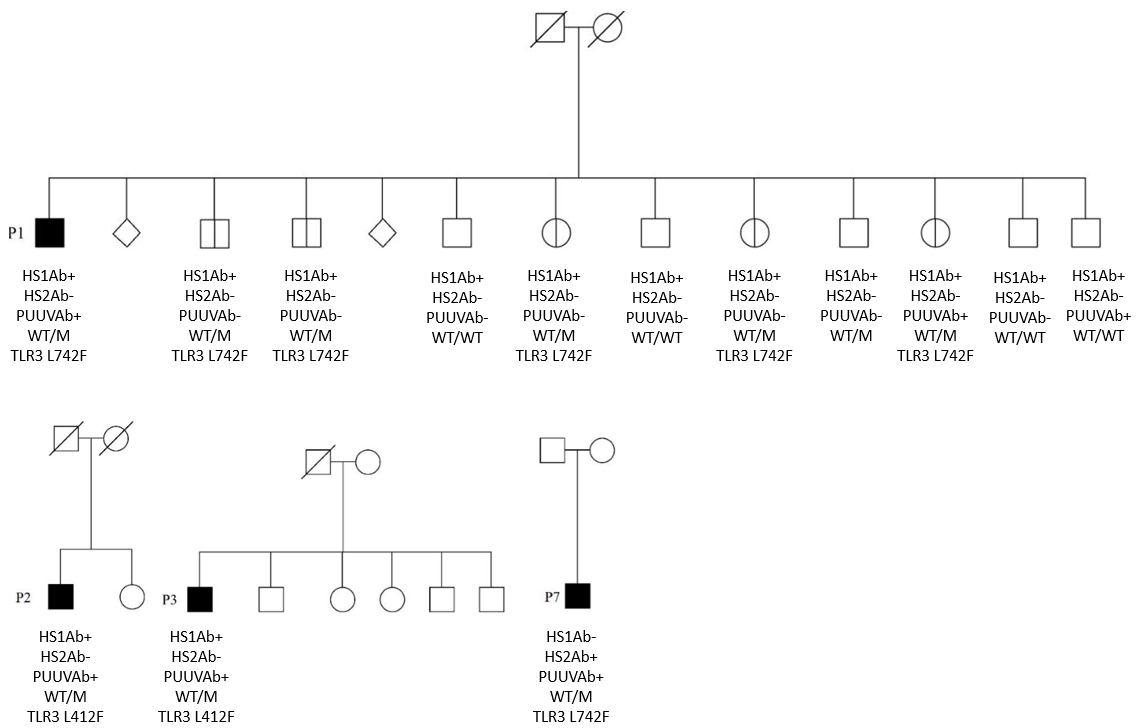


**Supplementary figure.** The family trees of patients with TLR3 p.L742F or p.L412F variants. We analyzed the family members of the patient 1 (P1) for TLR3 p.L742F variant and tested them for PUUV hantavirus, HSV1, and HSV2 serology; in addition to the index (P1), only one sibling was positive for TLR3 p.L742F variant, HSV1 IgG and PUUV hantavirus IgG. This family member had not suffered an episode of encephalitis. All family members were negative for HSV2 serology. Patients 2 and 3 with TLR3 p.L412F were positive for HSV1 antibodies and they had no history of HSE.

**Additional molecular genetics analysis:**

In addition to known Primary Immune Deficiency Disease (PIDD) and encephalitis genes, loss-of-function (LoF) variants of patients 1 and 7 were analyzed the whole exome wide. Allele frequency (MAF) >0.01 in Genome Aggregation Database (gnomAD; Cambridge, MA, USA; <https://gnomad.broadinstitute.org/>) were filtered out.  LoF variants identified frequently (more than 30 carriers or 15 homozygous individuals) in our cohort (480 exomes) were excluded from the analysis. Furthermore some LoF variants were excluded based on the Integrative Genomics Viewer visualization (IGV; <https://software.broadinstitute.org/software/igv/>), but none of the variants was confirmed by Sanger sequencing. The remaining LoF variants are listed in the Supplementary material Table E3.

Variant visualization on IGV did not show any remarkable regions of homozygosity revealing consanguinity in any of the patients in chromosome 4.

**Reference for the supplement**

1. Venkatesan A, Tunkel AR, Bloch KC, Lauring AS, Sejvar J, Bitnun A et al. Case definitions, diagnostic algorithms, and priorities in encephalitis: consensus statement of the international encephalitis consortium. Clin Infect Dis 2013;57:1114-1128.
